# Cardiac Stimulation for Suspended Animation by Direct Digital Manipulation1Read before the Surgical Section of the American Medical Association, Boston, Mass., June, 1906.

**Published:** 1906-07

**Authors:** Benjamin Merrill Ricketts

**Affiliations:** Cincinnati, O.


### Cardiac Stimulation for Suspended Animation by Direct Digital Manipulation.[1]

**Illustrated by Diagrams and Case Reports.**

A Supplementary Report to Surgery of The Heart and Lungs.

BY BENJAMIN MERRILL RICKETTS, M. D., LL. D., Cincinnati, O.

(*Author's Abstract.*)

THE definition of life is—The functions depending directly on the cerebrospinal nervous system and the voluntary muscles. The definition of death is—Cessation of the heart beat.

The various methods for determining death are considered, among them that of Foubert, who bases his sign of death upon this definition. He says, if the heart is found not to pulsate, dissolution has taken place. He introduces the finger through the intercostal space to the heart to determine this. With him the introduction of the finger was to condemn to death. Now the introduction of the finger is to restore life.

No known methods of determining death are positive in non-mutilated bodies within several minutes after cessation of the heart beat. For this reason all known methods for resuscitation should be employed when the heart ceases to beat until a time when positive methods of death can be applied.

Cardiac contraction has been induced many hours and even days after death had been made positive. Many lives have been unnecessarily sacrificed for the want of effort to save them.

1 Read before the Surgical Section of the American Medical Association, Boston, Mass., June, 1906.

No one method should be employed to the exclusion of all others, until more definite knowledge upon this subject is obtained by experimentation and observation upon the various forms of animals both living and dead. The case at Brighton, England, Nov. 11, 1905, is cited as an illustration to show the force of this argument. The child nine days old lived sixteen hours after the physician had pronounced it to be dead.

The class of cases in which rhythmic massage of the heart is appropriate are suspended animation due to ether, chloroform, nitrous oxide, carbon dioxide; shock due to fright, injury or surgical operation; drowning, electrocution, strangulation, loss of blood and probably diseases and drugs of many kinds. Experimental resuscitation by rhythmical cardiac compression was first practised by Schiff in 1874 and Ricketts in 1877. Defibrinated blood, sodium, adrenalin chloride, and Lock's solution are mentioned as of especial interest.

Hearts removed from both the live and dead body have been subjected to heat and cold, both dry and moist, por periods of time varying in length and found to contract when subjected to the electric current. The experiments of Westbrook 1882, Prus 1889, Velich 1896, Porter 1897, Ricketts 1899, Batelli 1900, Kuliabko 1902, Spina 1903, Crile 1903, D'Halluin 1904, Kemp and Gardener 1904, Muller 1905, with others, have shown that contractions of the heart have been induced in many ways under many abnormal circumstances.

It has been shown that lack of potassium salts renders the vagus wholly or nearly ineffective and the increase of calcium has no effect on vagus control. Intrathoracic transpericardial cardiac massage, was first done by Niehaus in 1880. It is done after making a horseshoe incision through the cutaneous structure and dividing the third, fourth and fifth ribs from right to left or left to right, preferably turning the flap from right to left. The hemorrhage is insignificant unless the internal mammary artery is injured. The pericardium is exposed and divided that the heart may be grasped between the thumb and forefinger. Care should be taken not to open the pleural cavity but if this has been done the opening in the pleura should be disregarded.

Of the twenty-one cases reported two were complete recoveries, eleven lived from one minute to twenty-seven hours, and in nine the pulse never returned. In nineteen the chest was deliberately opened; in two the chest was already opened. In sixteen chloroform was used and in four not stated. In one recovery the heart did not beat for three minutes, while in another it did not beat for one minute. Of those that died, in one the heart did not pulsate for sixty seconds, then lived for twenty-seven hours before dissolution. In two others the heart did not beat for fifteen minutes; one lived sixteen hours before final dissolution.

Subdiaphragmatic transperitoneal cardiac massage is supposed to have been first done by Starling and Land in 1902. The heart is grasped with the hand with the diaphragm intervening. Eighteen have been treated in this way, in eight of which the abdomen was deliberately opened for the purpose and in ten the abdomen had already been opened for other purposes; in three chloroform was used, in ten not stated; in three the diaphragm was deliberately opened from below to massage the heart. Ten permanently recovered, eight died, in five of which the pulsations were restored for a short time, and in three the pulsations were not restored. Of the eight in which the chest was deliberately opened four recovered, and of eight cases in which the abdomen had already been opened six recovered. Of the ten recoveries, in one the heart ceased to beat one minute, one four minutes, one ten minutes, one, one minute plus the time required to open the abdomen, and in seven not stated.

The subject is presented in five chapters—namely:

I Preamble.

II Experimental.

III Intrathoracic transpericardial.

IV Subdiaphragmatic Transperitoneal massage, and

V Miscellaneous bibliography.

Total cases operated 39.

Primary opening of chest alone 19, already open 1.

Primary opening of abdomen alone, 8, already open 9.

Opening of chest and abdomen 2.

Number of chest operations recovered 2-10%.

Number of abdominal operations recovered 10-62½%.

Number of diaphragms incised 3, all died.

Eight abdomens deliberately opened, 3 recovered.

Nine abdomens already opened, 7 recovered.

Number of males 16, females 7, not stated 16.

Number of chloroform administrations 27, ether 1, not stated 11.

Number of cases in which the character of operation for which the anésthetic was given 25, not stated 14.

Total number of cases in which the heart beat was reestablished 31.

Total number in which the heart beat was not reestablished 8.

Total number of cases in which the heart beat was permanently reestablished 12.

Number of ages given 22.

Time before heart beat was reestablished, one to sixty minutes.

Greatest length of time of cessation of heart beat to be reestablished permanently was 20 minutes.

